# Influence of Centrifugation and Transmembrane Treatment on Determination of Polyphenols and Antioxidant Ability for Sea Buckthorn Juice

**DOI:** 10.3390/molecules28062446

**Published:** 2023-03-07

**Authors:** Dan Wu, Qile Xia, Huilin Huang, Jinhu Tian, Xingqian Ye, Yanbin Wang

**Affiliations:** 1 National-Local Joint Engineering Laboratory of Intelligent Food Technology and Equipment, Zhejiang Key Laboratory for Agro-Food Processing, Integrated Research Base of Southern Fruit and Vegetable Preservation Technology, Zhejiang International Scientific and Technological Cooperation Base of Health Food Manufacturing and Quality Control, Fuli Institute of Food Science, College of Biosystems Engineering and Food Science, Zhejiang University, Hangzhou 310058, China; 2 Key Laboratory of Post-Harvest Handling of Fruits, Food Science Institute, Zhejiang Academy of Agricultural Sciences, Hangzhou 310021, China; 3 Zhejiang Academy of Forestry, Hangzhou 310023, China

**Keywords:** DPPH, ABTS, FRAP, centrifugation, transmembrane

## Abstract

When the total phenolic content (TPC) and antioxidant activity of sea buckthorn juice were assayed by spectrophotometry, the reaction solutions were not clarified, so centrifugation or membrane treatment was needed before determination. In order to find a suitable method for determining TPC and antioxidant activity, the effects of centrifugation and nylon membrane treatment on the determination of TPC and antioxidant activity in sea buckthorn juice were studied. TPC was determined by the Folin-Ciocalteau method, and antioxidant activity was determined by DPPH, ABTS, and FRAP assays. For Treatment Method (C): the sample was centrifuged for 10 min at 10,000 rpm and the supernatant was taken for analysis. Method (CF): The sample was centrifuged for 10 min at 4000 rpm, filtered by Nylon 66 filtration membranes with pore size of 0.22 μm, and taken for analysis. Method (F): the sample was filtered by Nylon 66 filtration membranes with pore size of 0.22 μm and taken for analysis. Method (N): after the sample of ultrasonic extract solution reacted completely with the assay system, the reaction solution was filtered by Nylon 66 filtration membranes with pore size of 0.22 μm and colorimetric determination was performed. The results showed that centrifugation or transmembrane treatment could affect the determination of TPC and antioxidant activity of sea buckthorn juice. There was no significant difference (*p* > 0.05) between methods (CF) and (F), while there was a significant difference (*p* < 0.05) between methods (C) (F) (N) or (C) (CF) (N). The TPC and antioxidant activity of sea buckthorn juice determined by the four treatment methods showed the same trend with fermentation time, and the TPC and antioxidant activity showed a significant positive correlation (*p* < 0.05). The highest TPC or antioxidant activity measured by method (N) indicates that method (N) has the least loss of TPC or antioxidant activity, and it is recommended for sample assays.

## 1. Introduction

Plant polyphenols are secondary metabolites of plants and are widely distributed in the roots, bark, leaves, and fruits of plants. As an active ingredient, polyphenols have potential health effects, including antioxidant, anti-inflammatory, anti-cancer, and other physiological functions [1,2,3,4,5,6]. It is one of the research hotspots in plant food [1,3,5,6,7].

The detection of plant polyphenols is very extensive, and the accuracy of the detection method is very important. Methods for the determination of polyphenolic compounds include spectrophotometry [7], high performance liquid chromatography (HPLC) [8,9], high performance liquid chromatography-mass spectrometry [10], gas chromatography (GC), and gas chromatography-mass spectrometry (GC-MS) [11,12]. Among them, spectrophotometry is the most commonly used method for the determination of TPC, including the Folin-Ciocalteu (F-C) assay, the Prussian Blue (P-B) assay, and the Ferrous Tartrate (F-T) assay. Gao et al. studied the influence of different methods (F-C, P-B and F-T) and standards on the determination of TPC, and the results indicated that different methods and standards of different structures have a great influence on the determination of total phenol contents [13]. The Folin-Ciocalteu (F-C) assay is the most common method for the determination of TPC at present [7,14,15].

Antioxidant activity is one of the efficacies of plant polyphenols. The evaluation methods of antioxidant activity include in vivo evaluation and in vitro evaluation. In vivo evaluation includes animal experiments and human experiments, in vitro evaluation includes DPPH assays, ABTS assays, FRAP assays, ORAC assays, TRAP assays, TOSC assays and so on [16,17,18,19,20]. Th in vivo evaluation system is close to the biological environment. It is sensitive, but the experimental period is long, the cost is great, and the operation is cumbersome. Therefore, the in vitro experimental evaluation system has been favored by scholars. DPPH, ABTS and FRAP assays are the most commonly used for the determination of antioxidant activity in vitro.

Sea buckthorn berries are rich in polyphenols, organic acids, and vitamins that may have antioxidant properties and have a positive effect on some diseases [21,22,23,24]. Ji et al. reviewed the antioxidant activity and mechanism of polyphenols from the *Hippophae* species, the polyphenols included 69 flavonoids, 15 phenolic acids, and 15 tannins; the main antioxidant activity mechanisms of the polyphenols were summarized as follows: regulating enzyme activity, affecting the antioxidant reaction of cells, lipid peroxidation, and free radical-scavenging activity [25]. The study of TPC and antioxidant activity are very important for sea buckthorn’s physiological activity. The Folin-Ciocalteu (F-C) assay for TPC and the three antioxidant activity determination methods (DPPH, ABTS and FRAP assay) for in vitro evaluation can be completed by using an UV spectrophotometer. The operation is simple and the instrument is common. Therefore, it is often used by scholars to initially evaluate the related physiological activity of food, and it is also common in the research of sea buckthorn-related products. These methods are used to determine the TPC and antioxidant activity of sea buckthorn here. However, sea buckthorn juice solution is opaque and orange, and when the TPC or antioxidant activity is determined by spectrophotometry, the reaction solution is cloudy and needs to be pretreated by centrifugation or transmembrane treatment. Some national standards or references have centrifugal or transmembrane treatment steps in the process of determining plant polyphenols [26,27,28,29]. Chen et al. used six different micro-filtration membranes (PES, Nylon, CA, PVDF, PTFE, and PP) to clarify bayberry juice, and the micro-filtration membranes of different materials had a significant effect on TPC and antioxidant activity (*p* < 0.05) [30]. However, there are no studies on the effect of centrifugation or membrane treatment on the determination of TPC or antioxidant activity of sea buckthorn juice. The effect of centrifugation or membrane treatment on the determination of TPC and antioxidant properties of sea buckthorn juice was studied here, which provides research guidance for the method for the determination of TPC and antioxidant properties of sea buckthorn juice.

## 2. Materials and Methods

### 2.1. Materials

Folin–Ciocalteu reagent and 6-hydroxy-2,5,7,8-tetramethyl-chroman-2-carboxylic acid (Trolox) were purchased from Shanghai yuanye Bio-Technology Co., Ltd. (Shanghai, China). 2,2-diphenyl-1-picrylhydrazyl (DPPH) and gallic acid were purchased from Shanghai Macklin Biochemical Co., Ltd. (Shanghai, China). 2,2′–azino-bis-(3-ethylbenzo-thiazoline-6-sulfonic acid) diammonium salt (ABTS) and 2,4,6-tri(2-pyridyl)-1,3,5-triazine (TPTZ) were purchased from Aladdin Industrial Corporation (Shanghai, China). Ethanol absolute, methanol anhydrous, sodium carbonate, potassium persulfate, glacial acetic acid, sodium acetate, acetic acid, iron trichloride, and hydrochloric acid (HCL) were purchased from Sinopharm Chemical Reagent Co., Ltd. (Shanghai, China).

Sea buckthorn (named “shengqiuhong”, *Fructus Hippophae*, produced in Tacheng, Xinjiang, China) was provided by Gansu Aikang Sea buckthorn Products Co.,Ltd. (Gansu, China). *Lactobacillus paracasei* was provided by Xi’an Jushengyuan Biotechnology Co., Ltd.(Xi’an, China). *Saccharomyces cerevisiae* was provided by Angelyeast Inc.(Hubei, China). Sucrose was from the local market.

### 2.2. Sample Preparation

Sea buckthorn was cleaned with sterile water, 500 g was taken into the DS-1 Homogenizer (Shanghai Specimen and Model Factory, Shanghai, China) to make sea buckthorn juice, and then put into the fermentation tanks (sterilization). 500 g of sterile water, 50 g of sugar, 0.75 g of *Saccharomyces cerevisiae*, 2.5 g of *Lactobacillus paracasi* were added into the sea buckthorn juice and mixed uniformly. Under anaerobic conditions, the sea buckthorn juice fermented for 0, 1d, 2d, 3d, 4d, 5d, 6d in a 37 °C incubator. The samples were filtered by 400 mesh filter cloth, 1 mL of each sample was taken into 4 mL ethanol absolute and mixed respectively. The mixture was thoroughly extracted for 30 min by a SK1200B ultrasonic extractor (Shanghai Kedao Ultrasonic Instruments Co. Ltd., Shanghai, China) and ready for use.

### 2.3. Centrifugation and Transmembrane Treatment

Method (C): The sample of ultrasonic extract solution was centrifuged for 10 min at 10,000 rpm and ready for analysis.

Method (CF): The sample of ultrasonic extract solution was centrifuged for 10 min at 4000 rpm, filtered by Nylon 66 filtration membranes with pore size of 0.22 μm and ready for analysis.

Method (F): The sample of ultrasonic extract solution was filtered by Nylon 66 filtration membranes with pore size of 0.22 μm and ready for analysis.

Method (N): After the sample of ultrasonic extract solution reacted completely with the assay system, the reaction solution was filtered by Nylon 66 filtration membranes with pore size of 0.22 μm and colorimetric determination was performed.

### 2.4. Determination of Total Phenolic Content (TPC)

TPC standard solution (ρ = 1000 mg/L): gallic acid was weighed at 0.1000 g, dissolved in methanol anhydrous, transferred to a 100 mL volumetric bottle, and diluted with methanol anhydrous to volume.

Standard solution for TPC (ρ = 0, 40, 80, 120, 160, 200, 240 mg/L): 0, 0.4, 0.8, 1.2, 1.6, 2.0, and 2.4 mL of the TPC standard solution (ρ = 1000 mg/L) were transferred into 10 mL volumetric flasks with a pipettor (Thermofifisher Scientifific Co., Ltd., Waltham, MA, USA) and diluted with methanol anhydrous to volume.

Preparation of the TPC standard curve: 400 μL of the standard solution was mixed with 2.4 mL distilled deionized water and oxidised with 0.4 mL of 1 mol/L Folin–Ciocalteu reagent. The mixture was kept in the dark for 6 min at room temperature. Next, 1.6 mL of 10.5% Na_2_CO_3_ (*w*/*v*) was added, mixed, and reacted at room temperature for 60 min. Finally, 5.2 mL distilled deionized water was added and mixed. The absorbance of the mixture was measured at 760 nm by a DR3900 colorimeter (Hach Company, Loveland, CO, USA). Results were expressed as gallic acid equivalents (GAE) (mg GAE/L).

Sample determination: for the samples treated by method (C), (CF), and (F), the determination steps were the same as the preparation of the TPC standard curve; for the samples with no treatment, the reaction steps before colorimetry were consistent with the standard curve. After the reaction between the sample and reagent was over, the mixture was filtered by Nylon 66 filtration membranes with pore size of 0.22 μm and then was measured at 760 nm (Method (N)).

### 2.5. DPPH Assay

Trolox standard solution (ρ = 1000 mg/L): Trolox was weighed at 0.1000 g, dissolved in ethanol absolute, transferred to a 100 mL volumetric bottle, and diluted with ethanol absolute to volume.

Standard solution for Trolox (ρ = 0, 25, 50, 100, 150, 200 mg/L): 0, 0.25, 0.5, 1.0, 1.5, and 2.0 mL of the Trolox standard solution (ρ = 1000 mg/L) were transferred into 10 mL volumetric flasks with a pipettor (Thermofifisher Scientifific Co., Ltd., Waltham, MA, USA) and diluted with ethanol absolute to volume.

Preparation of DPPH-scavenging ability standard curve: 100 μL of the standard solution was added into 3.9 mL of 40 mg/L DPPH solution (dissolved in ethanol absolute), mixed in a vortex, reacted in the dark at room temperature for 30 min. The absorbance of the mixture was measured at 517 nm by a DR3900 colorimeter (Hach Company, Loveland, CO, USA).

DPPH-scavenging ability was calculated by the following formula:

$$\mathrm{DPPH}-\mathrm{scavenging}\;\mathrm{ability}\left(\%\right)=\frac{100\times \left(A0-\mathrm{An}\right)}{A0}$$

where A0 was the absorbance value of blank tube and An was the absorbance value of sample tube.

The standard curve of DPPH-scavenging ability vs. Trolox concentration was plotted. The results were expressed as Trolox equivalents (TE) (mg TE/L).

Sample determination: for the samples treated by method (C), (CF) and (F), the determination steps were the same as the preparation of DPPH-scavenging ability standard curve; for the samples with no treatment, the reaction steps before colorimetry were consistent with the standard curve. After the reaction between the sample and reagent was over, the mixture was filtered by Nylon 66 filtration membranes with pore size of 0.22 μm and then was measured at 517 nm (Method (N)).

### 2.6. ABTS Assay

ABTS+ cation solution: weighed ABTS 200 mg, potassium persulfate 34.4 mg, dissolved in 50 mL distilled deionized water, mixed in a vortex, reacted in the dark at room temperature for 24 h.

Preparation of ABTS-scavenging ability standard curve: the ABTS+ cation solution was diluted with ethanol absolute until it reached an absorbance measurement of 0.7 ± 0.02 at 734 nm. 100 μL of the Trolox standard solution (ρ = 0, 25, 50, 100, 150, 200 mg/L) was added into 3.9 mL of the diluted ABTS+ cation solution, mixed in a vortex, reacted in the dark at room temperature for 10 min. The absorbance of the mixture was measured at 734 nm by a DR3900 colorimeter (Hach Company, Loveland, USA).

ABTS-scavenging ability was calculated by the following formula:

$$\mathrm{ABTS}-\mathrm{scavenging}\;\mathrm{ability}\left(\%\right)=\frac{100\times \left(A0-\mathrm{An}\right)}{A0}$$

where A0 was the absorbance value of blank tube and An was the absorbance value of sample tube.

The standard curve of ABTS-scavenging ability vs. Trolox concentration was plotted. The results were expressed as Trolox equivalents (TE) (mg TE/L).

Sample determination: for the samples treated by method (C), (CF) and (F), the determination steps were the same as the preparation of ABTS-scavenging ability standard curve; for the samples with no treatment, the reaction steps before colorimetry were consistent with the standard curve. After the reaction between the sample and reagent was over, the mixture was filtered by Nylon 66 filtration membranes with pore size of 0.22 μm and then was measured at 734 nm (Method (N)).

### 2.7. FRAP Assay

Buffer solution (pH ≈ 3.6): weighed sodium acetate 5.10 g, dissolved in 100 mL distilled deionized water, added 20 mL of acetic acid, mixed and transferred into a 250 mL volumetric flask, diluted the mixture with distilled deionized water to volume.

TPTZ solution (0.01 mol/L): weighed TPTZ 312.3 mg, dissolved in 100 mL HCL solution (0.04 mol/L).

Iron trichloride solution (0.02 mol/L): transferred 10 mL HCL into 50 mL distilled deionized water, added iron trichloride 324.4 mg, mixed and transferred into a 100 mL volumetric flask, diluted the mixture with distilled deionized water to volume.

Preparation of the FRAP standard curve: the FRAP solution was prepared with buffer solution (pH ≈ 3.6), TPTZ solution (0.01 mol/L), and iron trichloride solution (0.02 mol/L) at a ratio of 10: 1: 1 (*v*/*v*/*v*). 100 μL of the Trolox standard solution (ρ = 0, 25, 50, 100, 150, 200 mg/L) was added into 2.4 mL of the FRAP solution and 2.5 mL distilled deionized water, mixed in a vortex, reacted in the dark at room temperature for 30 min. The absorbance of the mixture was measured at 593 nm by a DR3900 colorimeter (Hach Company, Loveland, CO, USA). The results were expressed as Trolox equivalents (TE) (mg TE/L).

Sample determination: for the samples treated by method (C), (CF) and (F), the determination steps were the same as the preparation of FRAP standard curve; for the samples with no treatment, the reaction steps before colorimetry were consistent with the standard curve. After the reaction between the sample and reagent was over, the mixture was filtered by Nylon 66 filtration membranes with pore size of 0.22 μm and then was measured at 593 nm (Method (N)).

### 2.8. Statistical Analysis

The Data Processing System (DPS, Hangzhou Ruifeng Information Technology Co., LTD, Hangzhou, China) software v13.5 was applied to fix the experimental data and establish the mathematical model [31].

## 3. Results and Discussion

### 3.1. Standard Curve of TPC, DPPH-Scavenging Ability (DPPH-SA), ABTS-Scavenging Ability (ABTS-SA) and FRAP

**Figure 1:**
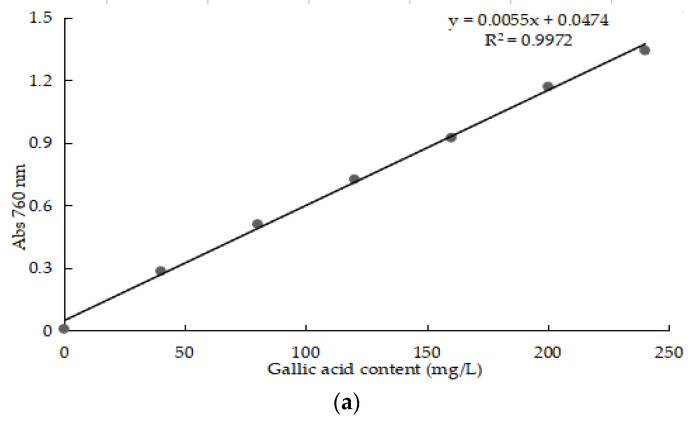

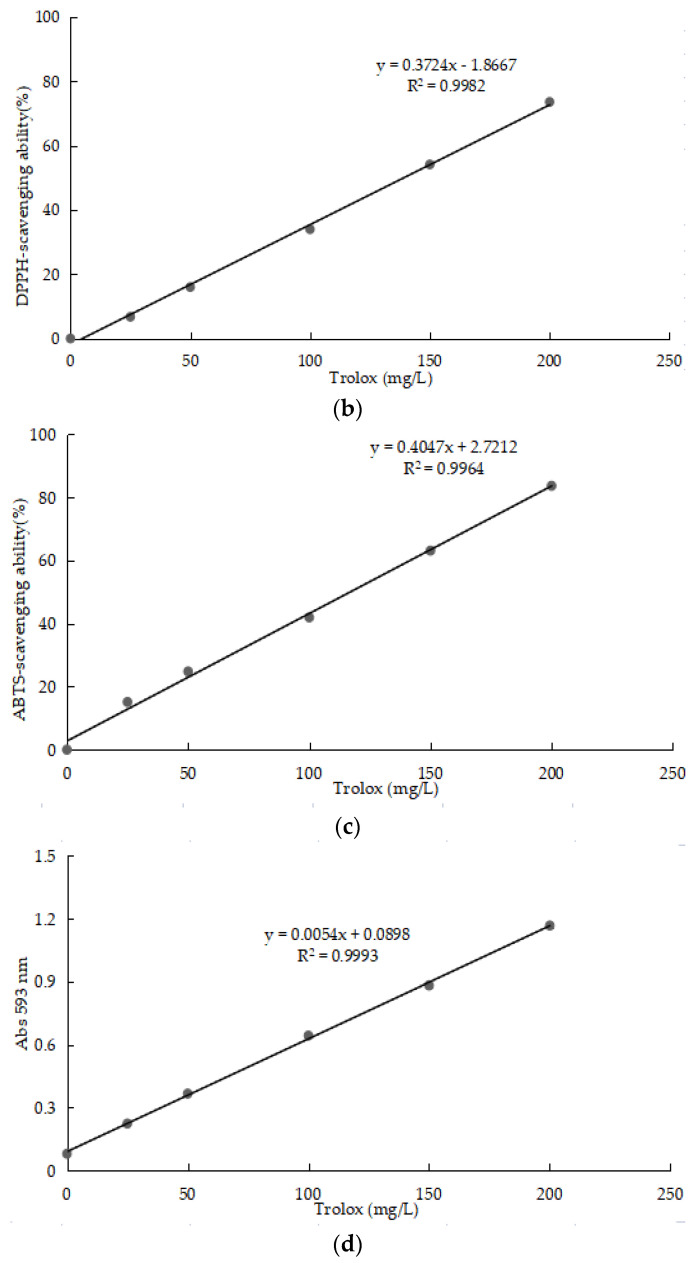
The standard curve of TPC, DPPH-SA, ABTS-SA and FRAP: (**a**)TPC standard curve; (**b**) DPPH-SA standard curve; (**c**) ABTS-SA standard curve; (**d**) FRAP standard curve.

The determination coefficient R^2^ of the standard curve of TPC, DPPH-SA, ABTS-SA and FRAP were all more than 0.995 and their linear regressions were good (Figure 1).

Although the Association of Official Analytical Chemists (AOAC) doesn’t set an official reagent-based method for TPC in foods, the F-C assay is highly popular. Different standard substances have great influences on the determination of TPC. Phenol, catechol, resorcinol, pyrogallic acid, gallic acid, tannic acid, proanthocyanidin, epicatechin (EC), epigallocatechin (EGC), and epigallocatechin Gallate (EGCG) were chosen as the standards in the determination of the TPC of tea by the F-C assay. The results showed that the TPC values determined by standard curve based on EGC, EGCG, and tannic acid were higher than gallic acid [13]. It was concluded that different hydroxyl groups affected the activity of the reaction, and the degree of polymerization affected the determination of TPC [13]. Martin et al. produced pomegranate polyphenols, and they found that it might be more accurate to estimate the TPC of POMx by using pomegranate polyphenols instead of gallic acid [32]. As sea buckthorn contains different polyphenols [22,24], it will be a new research topic to select a standard substance in TPC determination. DPPH, ABTS, and FRAP are common evaluation methods for sea buckthorn antioxidant ability [22,33,34]. The standard curve of DPPH-scavenging ability, ABTS-scavenging ability, and FRAP have a good correlation here. It will be used for sea buckthorn’s antioxidant ability.

### 3.2. Influence of Centrifugation and Transmembrane Treatment on Determination of Polyphenols

Platzer et al. showed that the F-C results depended on the number of fulfilled Bors criteria and the number of OH groups (if a molecule fulfills none of the Bors criteria) [35]. It was originally for the detection of tyrosine [36] and was later developed to measure the TPC [37]. Some fruit juices and their products were reported to need to be centrifuged or filtered before the determination of TPC, such as yellow peach wines that were centrifuged at 8000× *g* for 5 min before determination [38], sea buckthorn pulp that was centrifuged at 4000× r/min for 10 min [27], apple juice extraction that was filtered through a paper filter [8], mixed juice with blue honeysuckle juice and fermented goat milk that was centrifuged at 5000× *g* for 10 min [39], five kinds of smoothies including fruit and vegetables that were centrifuged at 5000× *g* for 5 min [40], fresh or fermented noni juice extracts that were filtered through a Nylon filter (0.22 µm, 25 mm) [41]. It is necessary to study the influence of centrifugation and filtration on the determination of polyphenols. Table 1 shows the changes in TPC in sea buckthorn juice determined by four methods. Table S1 shows variance analysis for TPC-determined methods in sea buckthorn juice. According to the same determination method, the TPC of sea buckthorn showed a gradually increasing trend with fermentation time. From Table 1 and Table S1, the results of method (CF) and method (F) have no significant differences (*p* > 0.05), but there are significant differences between other methods (*p* < 0.05). Methods (C), (CF) and (F) were all treated with membranes. The difference was that method © was centrifuged at a high speed at 10,000 revolutions per minute (rpm), method (CF) was centrifuged at a low speed at 4000 rpm, and method (F) was not centrifuged. It is seen that the centrifugation speed at 4000 rpm had no significant effect on the determination of TPC, and the centrifugation at 10,000 rpm had a significant effect on the determination of TPC. This phenomenon is mainly due to the positions of some phenolics under high-speed centrifugation. There are different effects of centrifugation for different fruit juices. Yousefnezhad, Mirsaeedghazi and Arabhosseini performed centrifugation on red beet and pomegranate juices at 2000 and 4000 rpm for 5 and 10 min [42]. They found that centrifugation had a negative effect on the polyphenol content of pomegranate juice, while centrifugation at 2000 rpm had a positive effect on the polyphenol content of red beet juice, compared to 4000 rpm which had a negative effect [42]. Jie et al. optimized an extraction method of neophenol-3-O-rutin from the Lilac flower, where 1000, 4000, 7000, and 10,000 rpm were selected to optimize the extraction and 4000 rpm/min had the largest content of neophenol-3-O-rutin [43]. It is beneficial to study the effect of centrifugal treatment on TPC before determination.

The main difference between methods (F) and (N) is whether the samples were treated by micro-filtration membranes before the chemical reaction. The result showed that, when the TPC was significantly reduced through micro-filtration membranes treatment before the chemical reaction, the reduction rate was about 17.51%—31.52% compared with micro-filtration membranes treatment after the chemical reaction. Different materials of micro-filtration membranes may have different micromorphology, resistance to acid-bases, and retention rates for different components. When Amirasgari and Mirsaeedghazi performed the filtration processing of red beet juice using a mixed cellulose ester membrane, they found that TPC, total soluble solid, color, and antioxidant activity was reduced by treatment [44]. Chen et al. used PES, Nylon, CA, PVDF, PTFE, and PP to filter bayberry juice, and the result showed that all membranes had negative effects on TPC, and the Nylon membrane had the greatest negative effect [32]. PVDF and PTFE had been proven to have good acid resistance and may be more suitable for the filtration of acidic juice [45].

### 3.3. Influence of Centrifugation and Transmembrane Treatment on Determination of DPPH-Scavenging Ability (DPPH-SA)

Table 2 shows the changes of DPPH-SA, ABTS-SA, and FRAP in sea buckthorn juice determined by four methods. Table S2 shows variance analysis for DPPH-, ABTS-, and FRAP-determined methods in sea buckthorn juice. From Table 2 and Table S2, the results of method (CF) and (F) have no significant differences (*p* > 0.05), but there are significant differences between other methods (*p* < 0.05). Methods (C), (CF) and (F) were all treated with membranes. The difference was that method (C) was centrifuged at a high speed at 10,000 revolutions per minute (rpm), method (CF) was centrifuged at a low speed at 4000 rpm, and method (F) was not centrifuged. It is seen that the centrifugation speed at 4000 rpm had no significant effect on the determination of DPPH-SA, ABTS-SA, and FRAP, and that centrifugation at 10,000 rpm had a significant effect on the determination of DPPH-SA. The decrease in antioxidant activity in sea buckthorn fruit juice by centrifugation may be mainly caused by the sedimentation of active antioxidant components, including phenolic compounds, carotenoids, tocopherols, tocotrienols and so on [22]. Polyphenols in sea buckthorn are mainly flavonoids and phenolic acids, there are 69 flavonoids, 15 phenolic acids, and 15 tannins [25]. The main difference between methods (F) and (N) is whether the samples were treated by micro-filtration membranes before the chemical reaction. The results showed that DPPH-SA, ABTS-SA, and FRAP were significantly reduced through micro-filtration membranes treatment before the chemical reaction, the reduction rate was about 3.97–7.53%(DPPH-SA), 18.86–24.40% (ABTS-SA), and 11.37–34.48% (FRAP) compared with micro-filtration membranes treatment after the chemical reaction. The decrease in antioxidant activity of sea buckthorn juice by micro-filtration membranes may be mainly caused by the interception of active antioxidant components, including phenolic compounds [33,46,47]. The membrane is a selective semi-permeable barrier between two phases, which certain components can pass while others cannot [46]. It was used for the purification of polyphenols and anthocyanins from natural sources [46,47]. According to the same determination method, the DPPH-SA, ABTS-SA, and FRAP of sea buckthorn showed a gradually increasing trend with fermentation time, which corresponds to the TPC trends.

### 3.4. Influence of Centrifugation and Transmembrane Treatment on Correlation of TPC and Antioxidant Ability

It has been reported that there is a correlation between TPC and antioxidant activity [30]. The research explores whether different treatments affect the correlation. From Table 3, it can be seen that all treatments have a significant correlation between each factor (*p* < 0.05). According to method (CF) and (F), there is a significant correlation at 0.05 level between TPC and DPPH-SA, and there are significant correlations at 0.01 level between TPC and ABTS-SA, TPC and FRAP, ABTS-SA and DPPH-SA, FRAP and DPPH-SA, FRAP and ABTS- SA.

The total phenol refers to a group of polyphenol substances, they are secondary plant metabolites, having a pivotal role in counteracting stress and contributing to the sensory system [48]. Nearly 100 polyphenol substances have been reported [25]. However, due to the differences in origin and varieties, the polyphenol monomers in different sea buckthorn are different. Lutin, kaempferol, quercetin, isorhamnetin, and catechin are the main and most common polyphenols in sea buckthorn [49,50]. The determination of polyphenol monomers is different from TPC, it is generally conducted by HPLC or HPLC-MS, which is suitable for the determination of samples with trace components. There is a large amount of dilution treatment before determination. The samples are generally not centrifuged but must be treated with membranes to ensure that the instrument pipeline is not blocked by the samples. However, the determination methods of total phenol and antioxidant activity are both spectrophotometry, centrifugation and transmembrane treatment may result in different errors. Therefore, it is difficult to compare the difference between the four different treatments for polyphenol monomers and the total phenolic content or antioxidant activity. Through the above research, we can conclude that we should pay attention to the uniformity of the treatment process of samples in the correlation study of TPC, antioxidant activity, and polyphenol monomers.

## 4. Conclusions

In summary, high-speed centrifugation or transmembrane treatment could affect the determination of TPC and antioxidant activity of sea buckthorn juice, as there were significant differences (*p* < 0.05). Low-speed centrifugation treatment had no significant difference compared with no centrifugation treatment (*p* > 0.05). There is a significant correlation between TPC and antioxidant activity under the four treatment methods. Method (N) has the least loss of TPC or antioxidant activity, however, it is rarely seen in practical application. Without centrifugal treatment, the membrane rate is slow and the efficiency is low. Therefore, membrane treatment after low-speed centrifugation is relatively more appropriate. High-speed centrifugation may lead to the sedimentation of macromolecular active substances, so it is not recommended. Of course, the changes in single-component active substances caused by membrane or centrifugation treatment shall be further explored and studied. Researchers need to choose different treatment methods according to their actual needs. Especially in the comparison of phenolic monomer substances, it is necessary to standardize the treatment method of the sample in monomer analysis and the TPC and antioxidant ability methods.

## Figures and Tables

**Table 1:** Changes of TPC in sea buckthorn juice determined by different methods.

Fermentation Time (d)		0	1	2	3	4	5	6
TPC (mg GAE/L)	Method (C)	596.44 ± 23.35 ^h^	608.77 ± 10.48 ^hgf^	618.12 ± 8.73 ^hgf^	631.03 ± 13.92 ^hgf^	630.54 ± 31.96 ^hgf^	630.57 ± 27.71 ^hgf^	633.58 ± 24.09 ^hgf^
	Method (CF)	602.95 ± 19.46 ^h^	612.12 ± 15.98 ^hgf^	622.94 ± 12.68 ^hgf^	641.46 ± 20.25 ^hgf^	661.35 ± 11.55 ^hgfe^	669.03 ± 17.11 ^hgfe^	689.64 ± 34.07 ^ef^
	Method (F)	606.61 ± 17.73 ^hg^	614.48 ± 26.35 ^hgf^	620.24 ± 14.50 ^hgf^	648.42± 42.76 ^hgf^	659.33 ± 25.03 ^hgfe^	662.67 ± 8.45 ^hgfe^	684.79 ± 20.99 ^gfe^
	Method (N)	735.39 ± 28.49 ^de^	810.55 ± 16.84 ^cd^	850.55 ± 20.25 ^bc^	875.70 ± 29.41 ^bc^	905.39 ± 16.50 ^b^	928.73 ± 28.88 ^ba^	999.94 ± 65.70 ^a^

Note: Different letters in the same row indicate statistically significant differences in the results (*p* < 0.05).

**Table 2:** Changes of DPPH-SA, ABTS-SA and FRAP in sea buckthorn juice determined by different methods.

Fermentation Time (d)		0	1	2	3	4	5	6
DPPH-SA (mg TE/L)	Method (C)	438.84 ± 17.83 ^h^	443.87 ± 20.78 ^hg^	452.05 ± 13.97 ^hg^	462.74 ± 36.65 ^hgf^	479.09 ± 15.44 ^hgfe^	472.8 ± 13.25 ^hgf^	480.98 ± 26.61 ^hgfde^
	Method (CF)	477.83 ± 20.00 ^hgf^	507.39 ± 10.50 ^bcdef^	532.54 ± 22.74 ^dabc^	537.58 ± 6.54 ^abc^	540.09 ± 15.82 ^abc^	541.35 ± 8.65 ^abc^	540.72 ± 12.28 ^abc^
	Method (F)	476.58 ± 8.92 ^hgf^	494.18 ± 14.73 ^gfcde^	531.92 ± 11.32 ^abcde^	534.43 ± 13.65 ^abc^	539.46 ± 14.97 ^abc^	543.24 ± 5.66 ^abc^	541.35 ± 11.78 ^abc^
	Method (N)	505.5 ± 7.55 ^bcdef^	534.43 ± 7.14 ^abc^	553.93 ± 4.75 ^ab^	571.53 ± 8.22 ^a^	579.08 ± 11.78 ^a^	576.57 ± 27.75 ^a^	582.85 ± 28.49 ^a^
ABTS-SA (mg TE/L)	Method (C)	453.60 ± 16.69 ^h^	565.21 ± 22.68 ^efgh^	602.41 ± 28.08 ^cdefg^	629.23 ± 10.49 ^cdefg^	645.67 ± 39.31 ^cdef^	640.48 ± 23.26 ^cdef^	641.35 ± 27.35 ^cdef^
	Method (CF)	511.02 ± 38.46 ^gh^	589.14 ± 20.59 ^defg^	601.87 ± 49.94 ^cdefg^	680.84 ± 41.88 ^cde^	686.79 ± 33.12 ^bcd^	696.98 ± 20.38 ^bcd^	703.77 ± 16.96 ^bcd^
	Method (F)	525.68 ± 23.56 ^fgh^	587.27 ± 39.90 ^defg^	612.09 ± 40.37 ^cdefg^	684.71 ± 31.00 ^bcde^	689.31 ± 25.02 ^bcde^	703.10 ± 38.74 ^bcd^	704.94 ± 44.12 ^bcd^
	Method (N)	682.63 ± 55.58 ^cde^	723.81 ± 72.09 ^bc^	809.61 ± 28.35 ^ab^	895.41 ± 48.08 ^a^	897.98 ± 25.74 ^a^	916.86 ± 22.49 ^a^	922.00 ± 27.76 ^a^
FRAP (mg TE/L)	Method (C)	407.59 ± 10.52 ^k^	445.86 ± 13.14 ^jk^	485.06 ± 32.08 ^efghijk^	523.02 ± 16.98 ^defghij^	533.83 ± 23.69 ^defghi^	536.91 ± 21.40 ^defghi^	548.33 ± 6.07 ^defg^
	Method (CF)	459.14 ± 6.17 ^hijk^	477.65 ± 5.58 ^fghijk^	545.86 ± 20.38 ^defgh^	560.99 ± 26.78 ^defg^	562.84 ± 33.67 ^def^	565.62 ± 18.55 ^de^	599.57 ± 17.18 ^cd^
	Method (F)	457.28 ± 47.97 ^ijk^	475.8 ± 14.05 ^ghijk^	552.65 ± 21.84 ^defg^	562.84 ± 16.31 ^def^	570.56 ± 15.30 ^de^	574.26 ± 13.04 ^d^	600.49 ± 21.96 ^cd^
	Method (N)	515.93 ± 11.82 ^defghij^	671.17 ± 38.00 ^bc^	722.10 ± 23.90 ^b^	732.90 ± 33.54 ^b^	851.11 ± 36.93 ^a^	844.32 ± 45.45 ^a^	916.54 ± 65.38 ^a^

Note: Different letters in the same row indicate statistically significant differences in the results (*p* < 0.05).

**Table 3:** Correlations of TPC, DPPH-SA, ABTS-SA and FRAP determined by different methods.

Method (C)		TPC	DPPH-SA	ABTS-SA	FRAP
	TPC	1			
	DPPH-SA	0.8838 **	1		
	ABTS-SA	0.9208 **	0.8500 *	1	
	FRAP	0.9491 **	0.9528 **	0.9488 **	1
Method (CF)		TPC	DPPH-SA	ABTS-SA	FRAP
	TPC	1			
	DPPH-SA	0.8032 *	1		
	ABTS-SA	0.9244 **	0.9342 **	1	
	FRAP	0.8957 **	0.9376 **	0.9216 **	1
Method (F)		TPC	DPPH-SA	ABTS-SA	FRAP
	TPC	1			
	DPPH-SA	0.8599 *	1		
	ABTS-SA	0.9410 **	0.9356 **	1	
	FRAP	0.9244 **	0.9780 **	0.9352 **	1
Method (N)		TPC	DPPH-SA	ABTS-SA	FRAP
	TPC	1			
	DPPH-SA	0.9352 **	1		
	ABTS-SA	0.9196 **	0.9791 **	1	
	FRAP	0.9777 **	0.9528 **	0.9136 **	1

Note: ** Significant correlation at 0.01 level; * significant correlation at 0.05 level.

## Data Availability

The data that support the findings of this study are available from the corresponding author upon reasonable request.

## Supplementary Materials

The following supporting information can be downloaded at: https://www.mdpi.com/article/10.3390/molecules28062446/s1, Table S1: Variance analysis for TPC determined methods in sea buckthorn juice; Table S2: Variance analysis for DPPH, ABTS and FRAP assay methods in sea buckthorn juice.

## Abbreviations

TPC	Total phenolic content
HPLC	High performance liquid chromatography
GC	Gas chromatography
GC-MS	Gas chromatography-mass spectrometry
F-C	Folin-Ciocalteu
G-B	Prussian Blue
F-T	Ferrous Tartrate
DPPH	2,2-diphenyl-1-picrylhydrazyl
ABTS	2,2′–azino-bis-(3-ethylbenzo-thiazoline-6-sulfonic acid) diammonium salt
FRAP	Ferric ion reducing antioxidant power
ORAC	Oxygen radical absorbance capacity
TRAP	Total peroxyl radical-trapping antioxidant parameter assay
TOSC	Total oxyradical scavenging capacity
DPPH-SA	DPPH-scavenging ability
ABTS-SA	ABTS-scavenging ability
PES	Polyether sulfone
CA	Acetate fiber
PVDF	Polyvinylidene fluoride hydrophilic
PTFE	Polytetrafluoroethylene hydrophilic
PP	Polypropylene membrane
EC	Epicatechin
EGC	Epigallocatechin
EGCG	Epigallocatechin Gallate
