# Supplementary material for: Influence of Centrifugation and Transmembrane Treatment on Determination of Polyphenols and Antioxidant Ability for Sea Buckthorn Juice
Source: Molecules. 2023 Mar 7;28(6):2446. doi: 10.3390/molecules28062446 (PMC10056822; doi:10.3390/molecules28062446)
Supplement: Supplementary file 1 [file molecules-28-02446-s001.zip › molecules-2153111-supplementary.pdf]

Supplementary material

Table S1. Variance analysis for TPC determined methods in sea buckthorn juice.

Sources of variation	F	ϱ
Method (C)VS.(CF)	11.155	0.0024
Method (C)VS.(F)	10.261	0.0185
Method (C)VS.(N)	835.531	0.0000
Method (CF)VS.(F)	0.004	0.9517
Method (CF)VS.(N)	731.807	0.0000
Method (F)VS.(N)	648.982	0.0000

Table S2. Variance analysis for DPPH, ABTS and FRAP assay methods in sea buckthorn juice.

Antioxidant activity	Sources of variation	F	ϱ
DPPH assay	Method (C)VS.(CF)	121.0550	0.0000
	Method (C)VS.(F)	126.1000	0.0000
	Method (C)VS.(N)	260.7010	0.0000
	Method (CF)VS.(F)	0.0010	0.9800
	Method (CF)VS.(N)	45.7960	0.0000
	Method (F)VS.(N)	62.7340	0.0000
ABTS assay	Method (C)VS.(CF)	13.0200	0.0012
	Method (C)VS.(F)	24.3680	0.0000
	Method (C)VS.(N)	205.7810	0.0000
	Method (CF)VS.(F)	0.1010	0.7520
	Method (CF)VS.(N)	190.5130	0.0000
	Method (F)VS.(N)	243.5980	0.0000
FRAP assay	Method (C)VS.(CF)	45.1330	0.0000
	Method (C)VS.(F)	43.2920	0.0000
	Method (C)VS.(N)	691.7160	0.0000
	Method (CF)VS.(F)	0.2080	0.6510
	Method (CF)VS.(N)	472.2840	0.0000
	Method (F)VS.(N)	422.8060	0.0000
